# Evaluation Methodology of a Smart Clothing Biomechanical Energy Harvesting System for Mountain Rescuers

**DOI:** 10.3390/s21030905

**Published:** 2021-01-29

**Authors:** Bartosz Pękosławski, Łukasz Starzak, Anna Dąbrowska, Grażyna Bartkowiak

**Affiliations:** 1 Department of Microelectronics and Computer Science, Lodz University of Technology, 90-924 Lodz, Poland; lukasz.starzak@p.lodz.pl; 2 Laboratory of Protective Clothing, Department of Personal Protective Equipment, Central Institute for Labour Protection—National Research Institute, 90-133 Lodz, Poland; andab@ciop.lodz.pl (A.D.); grbar@ciop.lodz.pl (G.B.)

**Keywords:** biomechanical energy, energy harvesting, electromagnetic transducer, electromagnetic generator, smart clothing, wearable device, power supply, voltage converter

## Abstract

The article presents a methodology developed for the evaluation of biomechanical energy harvesting systems that permits avoiding long-duration outdoor tests while providing realistic input signals and preserving uniform conditions across repeated tests. It consists of two stages: transducer output signal recording and power conversion and storage system measurements. The proposed approach was applied to assess the usefulness of a commercial electromagnetic transducer for supplying a Global Positioning System (GPS) receiver used as an active component of a smart clothing dedicated for mountain rescuers. Electrical power yield measurements have been presented together with ergonomic tests results. They all involved diverse physical activities performed by mountain rescuers that simulated their true operations, but were conducted in a training room for the sake of standardization. By providing reliable data on the transducer’s performance under realistic use conditions, the proposed evaluation procedure revealed that the true energy yield was much smaller not only with respect to the manufacturer’s assertions, but also substantially lower than what was expected based on an independent review which used unrealistic and non-uniform excitations. On the other hand, ergonomics ratings given by potential end users were very high, which demonstrates that the evaluated transducer can still be useful for supplying active cloth components with a lower power demand. The study also revealed that transducer location and orientation strongly affect its performance, which must be taken into account at the first stage of the evaluation procedure. Moreover, physical activity type and conditions (such as motion speed and ground tilt) influence the output power and should be carefully considered when composing a typical case scenario for the second stage.

## 1. Introduction

### 1.1. Energy Harvesting for Mountain Rescuer Active Clothing

Mountain rescue operations are often conducted for many hours by a group of rescuers who search for lost walkers or climbers. The rescuers are equipped with several electric and electronic devices including GPS receivers, torches, radio and cell phones. All of these devices are battery-powered and consume electrical energy whose amount may vary in time depending on the action. Unfavorable conditions (low air temperatures, weak signal power, low visibility) often occur and lead to short battery lifetimes. As a result, additional energy storage is required, which adds weight and also has limited capacity. By using energy harvesting, mountain rescuers could be provided with an additional, self-recharging power bank that reduces the necessary capacity of an emergency battery. In this context, solar energy, thermal energy and mechanical energy may be considered as potential energy sources for harvesting.

Solar energy can be harvested by photovoltaic (PV) generators. They offer high power density of the order of 10 mW/cm^2^ [1] and have been successfully used in recent wearable systems [2,3,4]. However, their integration with clothing usually requires the use of flexible PV modules, so as not to impede body movements by rigid components placed on clothing. The efficiency of flexible PV generators is lower (up to 10% for organic PV modules; 3–4% typically) as compared to standard monocrystalline cells [5]. Moreover, the amount of harvested power strongly depends on insolation conditions (even a tenfold lower power density for a lightly cloudy sky) as well as module position and temperature. When a rescue operation is performed after dark or when weather conditions deteriorate (which is often the case in the mountains), other energy harvesting sources become more reliable.

Thermal energy can be harvested by thermoelectric generators (TEGs) based on Peltier modules. The studies where their potential use for wearable systems is analyzed [6,7] have shown that TEGs offer very low power densities in the order of 10 µW/cm^2^. This is related to their low conversion efficiency, low temperature gradients when placed on human body and very low output voltages of the modules. In order to maximize the power output of a TEG one would need to use thick and large modules with bulky heat sinks, in a direct contact with the skin on one side and air flowing on the other. This would cause discomfort for the user, related to skin cooling and motion impediment. This is why, when TEGs are integrated with clothing, no direct contact with skin exists and power density is as low as 2.6 nW/cm^2^ to 6.5 nW/cm^2^ [8].

Mechanical energy of human motion (also called biomechanical energy) can be harvested in various ways. The most common methods described in literature are based on piezoelectric, triboelectric, reverse electrowetting on dielectric (REWOD) and electromagnetic phenomena. Piezoelectric generators can be installed in shoe soles [9,10,11], yielding power densities of up to 10 µW/cm^2^, in backpack shoulder straps [12] (1.1 µW per gram of backpack load) or as fibers in textiles (30 µW per gram of fiber). Much higher power levels (up to 0.9 mW/cm^2^) can be achieved when using triboelectric textiles, which harvest energy from both human motions and wind gusts [13,14]; the problems related to their application are high output voltages (in the order of several hundred volts) and low output currents (much below 1 mA), resulting in high optimum load resistances. REWOD-based generators [15] can provide power densities as high as 0.8 mW/cm^2^, but require an external voltage source and a high-frequency excitation by self-oscillations while their design requires the bubble growth and collapse process to be modelled. Electromagnetic (EM) transducers can offer the highest power densities and may have various forms, including heel-strike devices [16], orthopedic knee braces with rotary generators [17], linear generators installed on arms or legs [18], or rotary generators moved by vertical movements of a backpack load with respect to its frame [19]. Depending on the electromagnetic generator type and size, their output power density from walking can reach 4.4 mW per gram of generator weight [17] or 2 mW per gram of backpack load mass [19].

The main problem associated with the application of many electromagnetic transducers is the fact that they cannot be easily integrated with clothing and may be inconvenient for the user because of their size and weight. The most appropriate for typical applications, including mountain rescue operations, are small linear generators that can be installed on a user’s leg or arm. These transducers are lightweight and can be easily mounted on or in clothing. However, they offer lower power outputs (on the order of dozens or hundreds milliwatts) than other types of electromagnetic generators (whose power may reach 10 W). Therefore, their performance must be reliably evaluated before they are applied for a specific purpose.

### 1.2. Electromagnetic Harvester Evaluation

The work described in this article was related to the design of a smart clothing for mountain rescuers which was supposed to include, among others, a GPS localization feature to track the rescuer as well as to map the area searched for better coordination. This function can be provided by standard smartphones, but their power demand is high, which makes them prone to battery discharge, especially in cold conditions. Therefore, it was decided to investigate the possibility of using a standalone GPS receiver with a dedicated energy source.

As demonstrated in an earlier study, power densities offered by flexible solar cells are sufficient for this application and the latter are easily integrated with clothing [20]. Nevertheless, solar power was rejected due to the likely unavailability or insufficient intensity of sunlight at night or in a shaded terrain. On the other hand, power outputs of thermoelectric generators are much too low. Therefore, an electromagnetic transducer seemed the best choice.

Commercially available electromagnetic biomechanical energy harvesters lack detailed technical data, which makes it difficult to make an optimum choice without appropriate testing. However, evaluation methodologies proposed so far have significant drawbacks.

During the tests described in [21], a generator was not attached to the human body. Instead, excitation was applied by shaking it with a large amplitude while holding the device in the hand. This was unnatural, thus unreliable; as well as impossible to standardize, thus to compare against other generators. It also resulted in an excitation frequency of 4.8 Hz, which is greater than observed with human movements while walking or running [22]. Moreover, the power delivered to the battery was estimated by measuring the RMS value of the current, which is incorrect considering that battery voltage is essentially constant over the time interval concerned.

A better approach was taken in [18], where movements were generated by a custom machine. This made them more uniform, still not entirely repeatable, as admitted by the authors. Nevertheless, the nature and the direction of this motion stayed artificial.

In the tests described in [17] and [19], human volunteers were exercising on a treadmill, which resulted in realistic movements of the oscillating mass and uniform test conditions. However, just a single activity type, walking on a flat horizontal surface, and a single speed were investigated, which was far from a mountain rescuer’s typical behavior on a mission. Moreover, this approach still limits the credibility of comparisons between different transducers, as it is impossible for persons to exactly reproduce their movements from the past.

Therefore, a need exists for an evaluation methodology that would offer high result credibility by combining:

(1) realistic inputs for the power conversion and storage subsystem;
(2) uniform conditions for different test participants;
(3) repeatability for various systems, their configurations or parameters;
(4) minimum time load on the participants.

This article aims at proving that a hybrid, two-stage testing methodology that involves short-time standardized physical exercises performed by human volunteers at a first stage and the use of an electronic model over long time intervals at a second stage can successfully address these requirements. Its usefulness will be demonstrated for a thorough, standardized testing procedure conducted in laboratory. In addition, its validity will be proven by comparing results yielded by the model to data obtained in real terrain under conditions that could be kept as close to uniform as possible.

## 2. Materials and Methods

### 2.1. System Mathematical Description

**Figure 1:**
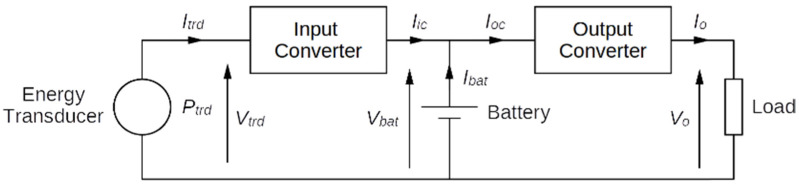
Schematic diagram of a typical mechanical energy harvesting system.

A typical system mechanical energy harvesting system is composed of an energy transducer, a rechargeable battery, a power processor and a load (Figure 1). The power processor is composed of an input converter, serving as an interface to the transducer, and an output converter, providing power to the load.

The load is supplied with a voltage *V_o_* and draws a current *I_o_*. The current *I_oc_* drawn by the output converter is:

(1) $$I_{oc}=\frac{V_{o}I_{o}}{\eta_{o}V_{bat}},$$

where *V_bat_* is the battery voltage and *η_o_* is the efficiency of the output converter. On the other hand, the current delivered by the input converter is:

(2) $$I_{ic}=\frac{\eta_{i}P_{trd}}{V_{bat}},$$

where *P_trd_* is the power generated by the transducer and *η_i_* is the efficiency of the input converter.

The current *I_ic_*, which results from energy harvesting, may be insufficient to cover the entire demand of the load. The remaining part must then be provided by the battery:

*I_bat_* = *I_oc_* − *I_ic_*. (3)

As the battery has some finite capacity *Q_bat_*, it will discharge in a time given by:

(4) $$t_{op}=\frac{Q_{bat}}{I_{bat}}=\frac{\eta_{o}Q_{bat}V_{bat}}{V_{o}I_{o}-\eta_{i}\eta_{o}P_{trd}},$$

which represents the operating time of the system. Without energy harvesting, *P_trd_* = 0, so the operating time would be shorter, equal to:

(5) $$t_{op0}=\frac{\eta_{o}Q_{bat}V_{bat}}{V_{o}I_{o}}.$$

Knowing both *t_op_* and *t*_*op*0_, one can determine the effective energy harvested, which is proportional to the output power and to the extension of the operating time:

*W_eh_* = *V_o_I_o_*(*t_op_* − *t*_*op*0_). (6)

The system operating time and the effective harvested energy are the ultimate indicators of a transducer’s performance and suitability for a given application, considering the load *I_o_* to be supplied and the available battery capacity *Q_bat_* (which usually results from cost, size or weight limits). As Equation (4) reveals, these parameters include the yield of the transducer in the system under consideration *P_trd_* as well as the effects of all the other components, represented by *V_bat_*, *V_o_*, *η_i_* and *η_o_*.

The output voltage *V_o_* is usually regulated at a given value. However, all the remaining parameters of Equation (4) may vary in time and may depend on each other. For instance, the output converter efficiency *η_o_* is a function of the output current *I_o_*. On the input side, the battery capacity *Q_bat_* depends on the battery current *I_bat_*, thus on the transducer power *P_trd_*; the battery voltage *V_bat_* depends on the instantaneous stored charge that results from *I_bat_*; in turn, the power delivered by the transducer *P_trd_* depends on the battery voltage *V_bat_* and it affects the efficiency *η_i_*. All these functions are non-linear.

Due to the non-linearity and the mutual dependence of the parameters involved, it is impossible to solve Equation (4) analytically so that to predict *t_op_*. On the other hand, solving it numerically would require several complex, multi-parameter relationships to be identified. For this reason, determining the operating time by experimental testing usually stays the only viable option.

### 2.2. Example Energy Harvesting System

To make the idea of the developed test methodology more clear, it will be presented based on the example of a specific energy harvesting system. As described in Section 1, its aim is to supply a GPS receiver integrated with clothing.

The selected GPS device was a GY-NEO6MV2 board from HiLetgo (Shenzhen, China) based on the NEO-6MV2 module from u-blox (Thalwil, Switzerland). Its average supply current is 17.6 mA at the supply voltage of 5 V, assuming that it would wake up every 1 min to check the location: the position is updated in 27 s with a supply current of 39 mA, while the standby current is 22 µA [23].

Based on the available information on commercially available solutions, the Ampy-Move electromagnetic transducer (Stryde Technologies, Evanston, IL, USA) was selected [24]. It is a linear generator with two fixed coils and moving permanent magnets mounted on springs.

The principle of the transducer operation is based on the Faraday’s law of induction, which states that an electromotive force (EMF) *E* is produced in an electric circuit by a time-varying magnetic field:

*E* = − *dΦ_B_*/*dt*, (7)

where *Φ_B_* is the magnetic flux enclosed by the circuit’s path. For electromagnetic transducers composed of tightly wound, single-layer solenoids with *N* identical turns of wire, the EMF becomes:

*E* = − *N* × *dΦ_B_*/*dt*. (8)

When a load is connected to the coil, an electric current *i* flows so that to produce a force *F_e_* that opposes the relative movement of the coil and the magnet inside the transducer. For a single-layer coil:

*F_e_* = − *Bli*, (9)

where *B* is the magnetic field, *l* is the single turn length.

**Figure 2:**
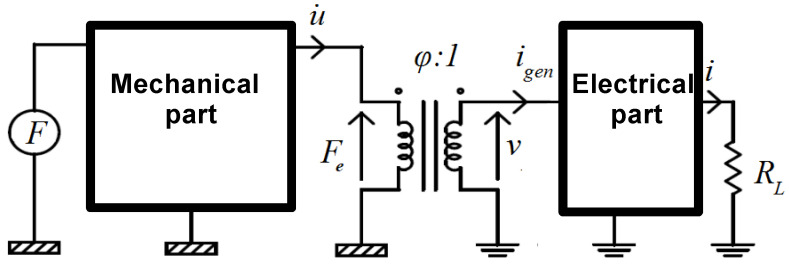
Lumped-element model of an electromagnetic transducer (*F* denotes the external mechanical force, *u*’ is the coil movement velocity, *ν* is the generated voltage, *i_gen_* is the generated electric current, *R_L_* is the load resistance).

A general lumped-element model (LEM) of an electromagnetic transducer consists of mechanical and electrical parts which are mutually coupled with a coupling coefficient *φ*, as shown in Figure 2.

The mechanical part can be attributed to all the mechanical phenomena inside the transducer, such as mechanical damping and elastic effects. The electrical part represent the transducer’s electric parameters, including parasitic ones. The coupling of both parts is responsible for both electric power generation and electric damping of movements. It should be emphasized that the determination of all the LEM parameters is difficult and model simulation may lead to substantial errors due to the simplified nature of the LEM.

**Figure 3:**
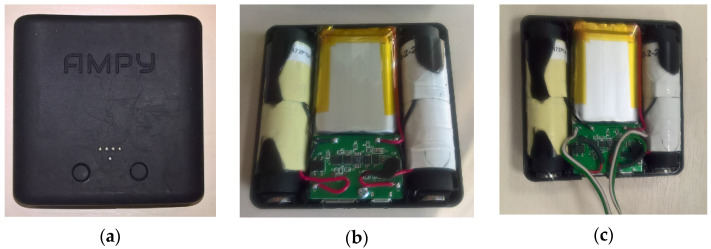
Ampy-Move generator: (**a**) outside view, (**b**) original inside view, (**c**) inside view after modifications.

The original Ampy-Move device, shown in Figure 3a,b, has an internal Li-ion battery and power conditioning circuits; its dimensions are 78 mm × 78 mm × 23 mm and its weight is 100 g. According to the manufacturer, it can be used as a power bank with a 5 V output and it is able to power a smartphone for 3 h from a 30-min running activity. However, no specific characteristics of this generator have been provided.

An independent test of Ampy-Move revealed that the true power output may be much smaller than asserted by the manufacturer [21]. Nevertheless, the author claims that the actual current delivered to the battery is approximately 20 mA. Supposing a battery voltage of 3.7 V (Li-ion battery) and an efficiency of 0.9, this would yield an output current of 13.3 mA at 5.0 V. Although this is too low to supply the GPS module continuously, the remaining energy can be provided by the battery; in order to obtain an operating time of 12 h, a capacity of 69.4 mAh would be sufficient.

It was not possible to measure the AC voltage generated by the transducer coils directly at the output of Ampy-Move, where a constant voltage of 5 V appears, produced by a power converter. It was therefore necessary to open the generator case, disconnect internal power processing circuits and connect two pairs of wires directly to the coils, as shown in Figure 3c.

According to measurements performed prior to the studies using the TiraVib S51110 electrodynamic shaker, the Ampy-Move transducer has a resonant frequency of approximately 9.5 Hz for an RMS excitation acceleration of 1.5*g*, with a 3 dB bandwidth approximately 5 Hz wide; the maximum power generated in these conditions reached 18.3 mW for a single coil. This means that Ampy-Move does not operate in mechanical resonance since the dominant human motion frequency components are in the range of 0.5 Hz to 5 Hz [22].

The measured coil resistance is 325 Ω and it is close to the optimum load resistance, at which the generator produces the highest power. The coil inductance of 68 mH has a minor influence on the generator’s output impedance, which is mainly resistive in the frequency range of interest. Ampy-Move coils cannot be coupled with each other neither in series or in parallel, as this would cause distortions of the generated voltage and a decrease in the generated power. Hence, a power processing circuit must have two independent inputs.

### 2.3. Power Processor

**Figure 4:**
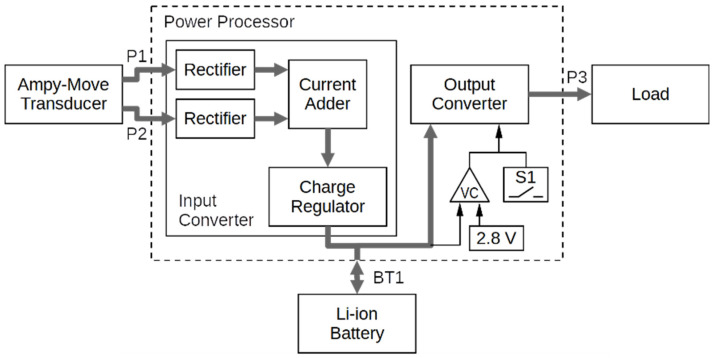
Block diagram of the power processing circuit (VC: Voltage Comparator).

**Figure 5:**
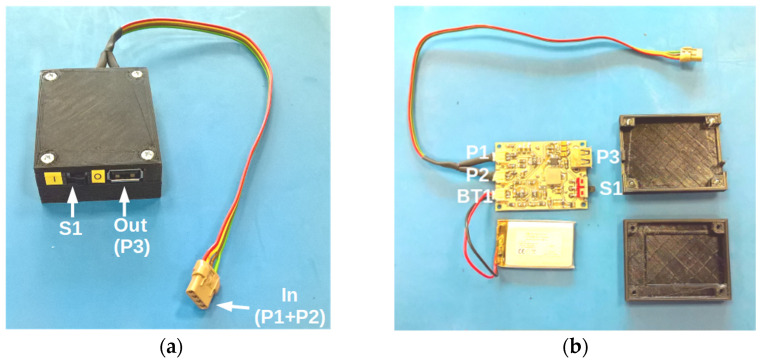
Power processing circuit with its input and output ports labelled (P1 and P2: input ports for the two Ampy-Move coils; P3: output USB port; BT1: battery connector; BT1: on/off button): (**a**) outside view, (**b**) inside view.

A block diagram of the power processor is shown in Figure 4. It is composed of an 85 mAh Li-ion battery; an input converter containing two full-wave rectifiers and a battery charging controller; and an output converter with a USB socket, providing a constant 5 V voltage and a maximum output current of 400 mA. Figure 5a presents an outside view of the power processing circuit case (whose dimensions are 73 mm × 51 mm × 21 mm) with its input and output sockets labelled, while Figure 5b presents the internal components of this circuit: the PCB and the battery.

The AC voltages from the transducer coils are rectified by diode bridge rectifiers and filtered by capacitors. Maximum input voltages allowed at the P1 and P2 ports are 40 V (peak-to-peak) and 10 V (rms). The current adder block (MOSFET-based dual “ideal diode” circuit LTC4415) let either coil charge the battery provided that the coil voltage is higher than the battery voltage. The battery charger is based on the LTC4071 integrated controller.

At the output side, the voltage comparator monitors the battery voltage and turns off the output converter if the voltage drops below 2.8 V, to protect the battery against excessive discharge (the minimum start voltage being 3.0 V). The output converter, based on the TPS61030 controller, boosts the battery voltage to approximately 5 V (the minimum output voltage measured is 4.5 V over the output current range of up to 400 mA). The S1 switch can be used to turn off the output converter to save battery charge.

**Figure 6:**
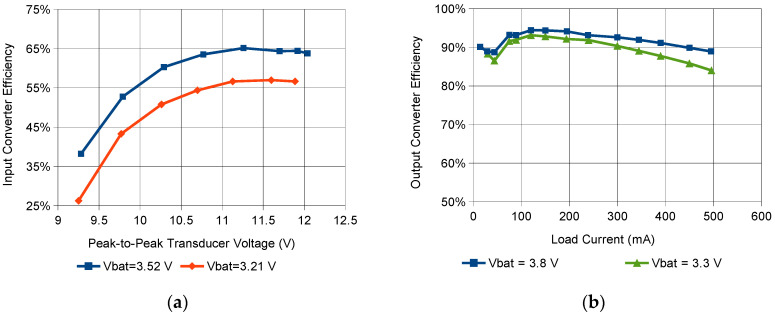
Efficiency characteristics of: (**a**) the battery charging block, (**b**) the output converter.

The battery charging block efficiency depends on both the battery voltage and the input voltage. It ranges from 26.3% to 65.1% when the battery voltage is between 3.2 V and 3.5 V and the peak-to-peak input voltage is between 9.2 V and 12.0 V; Figure 6a presents efficiency characteristics of this block. The charging block efficiency decreases for lower battery voltages as a result of larger input currents and higher power losses.

The output converter efficiency depends on both the output current and the battery voltage. It ranges between 82.1% and 94.4% for output currents from 14 mA to 400 mA when the battery voltage is between 3.3 V and 3.8 V. Figure 6b shows efficiency characteristics of this converter.

### 2.4. General Idea of the Evaluation Methodology

The principal aim of this study was to devise a methodology that would enable the performance and the suitability of a particular electromagnetic transducer to be determined for a specific active clothing component, as presented in Section 2.2. It follows from Section 2.1 that these properties are well described by the operating time *t_op_* and the corresponding effective harvested energy *W_eh_*. However, these parameters must be determined experimentally, which leads to the problems listed in Section 1. Moreover, such an assessment should cover various operating conditions of the system, i.e., motion activities of different type and intensity.

While devising an appropriate evaluation methodology, the following issues need to be considered to obtain the desired features as discussed in Section 1.

- Tests cannot be performed outdoors due to the impossibility to retain invariable conditions that affect human performance, e.g., temperature or humidity.
- Each experiment duration should be at least of the same order of magnitude as mountain rescuer work time during rescue operations, i.e., several hours. This would be a considerable physical and time load for volunteers, and it would cause random, unrepeatable variations of motion parameters due to different fatigue patterns.
- For a thorough assessment, it is necessary to repeat an experiment several times for various work conditions, while it is not possible to achieve invariant volunteer motion for several hours.

As a result, it was decided to develop a two-stage methodology:

(1) Measurement of the generator’s characteristics in laboratory, for several volunteers and for various work conditions (activities), resembling real ones. An ergonomics assessment can be simultaneously carried out at this stage.
(2) Measurement of the power conversion and storage subsystem (with a battery serving as a buffer between the generator and the load), also in laboratory, with the use of an electronic model that simulates the generator’s characteristics as measured at Stage 1.

This way, it becomes possible to perform repeated tests of system operation in the same conditions and for an identical motion sequence, over an arbitrary time. The two stages will now be described in the following two sections using the example of the particular energy harvesting system presented in Section 2.2 and Section 2.3.

### 2.5. First Stage of the Methodology

**Figure 7:**
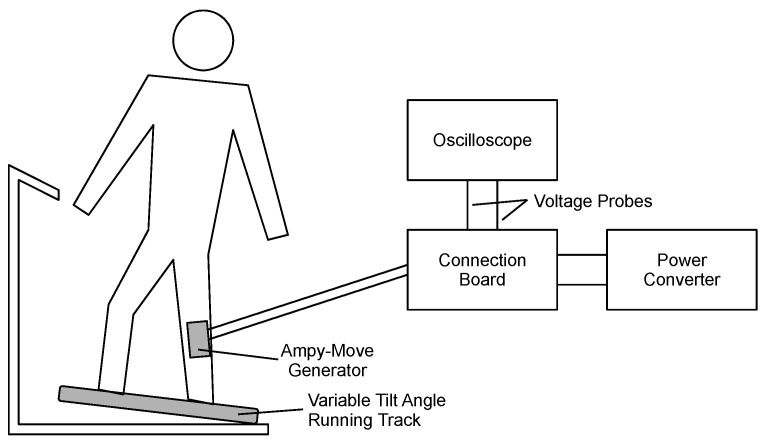
Laboratory setup for measurement of generator characteristics.

At the first stage of the procedure, the laboratory setup shown in Figure 7 was used. It consisted of:

- the Ampy-Move generator with the power processing circuit;
- an FDM-THM-M-3i running track (zebris Medical, Isny, Germany) with variable speed and tilt angle as well as a measurement module for gait parameters;
- a TPS2014B oscilloscope (Tektronix, Beaverton, OR, USA) with isolated channels and two Tektronix TPP0101 probes;
- a connection board for oscilloscope probes (custom-made).

Voltage waveforms at both generator coil outputs were measured simultaneously. The battery was fully discharged before measurements to achieve a maximum generator load and a maximum battery charging current. RMS values of coil voltages were then calculated for each activity. Since the voltages across the coils are generated at the instants when steps are made, the step frequency was also considered a test parameter. The latter was determined numerically as the fundamental frequency of the coil voltage from its FFT, by means of correlation.

The following procedure was followed while performing measurements at the first stage.

(1) Specific physical activity parameters were set according to Table 1.
(2) Volunteer movements were let settle down for approximately 30 s.
(3) Both coil voltage waveforms were recorded simultaneously for a defined time.
(4) Point 3 was repeated several times.

The time interval for recording voltage waveforms was chosen so as to achieve simultaneously:

- a time resolution of the oscilloscope waveform suitable to represent the fastest variations of voltage in time observed (the shortest time, the highest amplitude);
- waveform length corresponding to several dozen steps to limit the influence of accidental variations and volunteer movement irregularity.

It was observed that the optimum sampling interval for all types of motion was 10 ms, which corresponds to a measurement time of 25 s for the oscilloscope used, with a single waveform memory of 2500 samples. To meet the criterion of exact representation of transients, for single measurement intervals covering only a dozen or so strides, the recording was repeated several (at least three) times to achieve combined time intervals corresponding to about 50 steps. For each physical activity, additional parameters were recorded such as track reaction force for forefoot, metatarsus and heel, as well as step and stride lengths.

Five volunteers took part in these experiments, who agreed to participate after getting acknowledged with experiment details.

### 2.6. Second Stage of the Methodology

**Figure 8:**
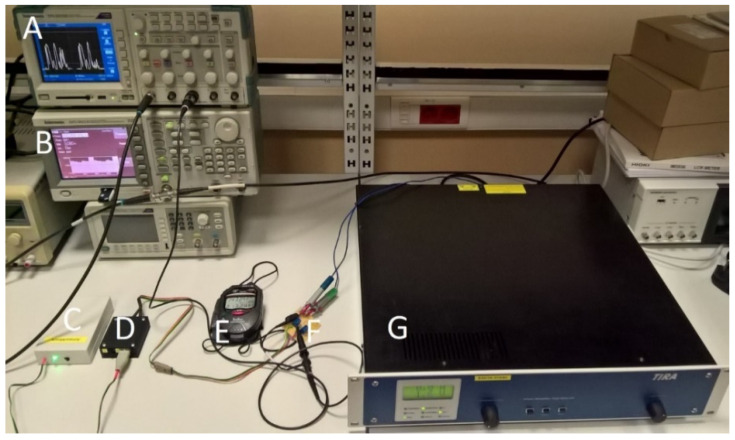
Laboratory setup for the energy harvesting system efficiency evaluation (**A**: oscilloscope, **B**: function generator, **C**: active component model load, **D**: power processing circuit, **E**: stopwatch, **F**: connection board, **G**: signal amplifier).

At the second stage of the procedure, the electromagnetic biomechanical energy transducer was replaced by an electronic model (shown in Figure 8) composed of:

- a Tektronix AFG 3021B arbitrary function generator with the ArbExpress software, which can generate voltage waveforms from recorded voltage samples (approximately 2000 for a single measurement);
- a TV 51,110 signal amplifier (TIRA, Schalkau, Germany) operating in its voltage mode, necessary to achieve output currents corresponding to those of the Ampy-Move transducer;
- the power processing circuit for the Ampy-Move transducer;
- a Tektronix TPS2014B oscilloscope and one Tektronix TPP0101 probe;
- a connection board for the oscilloscope probe (custom-made);
- an active component (GPS module) load model with a “no power” detector and sound signaling (custom-made);
- a Delta E 100 stopwatch (Hanhart 1882, Gütenbach, Germany).

In this setup, the operation time of the active component was measured with a constant load applied. Supply currents of real components often vary in time, usually in an unpredictable and unique manner. In order to repeat measurements in the same conditions both in respect of the input voltage (which is achieved by the generator model) and the load current, which also influences the measured operation time, the active component was replaced with its model built using a 280 Ω, 0.5 W resistor. The resulting constant load current was approximately 17.8 mA, which is close to the average supply current of the GPS module (17.6 mA), thus yielding an identical energy consumption.

To achieve coherent results in consecutive trials, the battery was fully charged at the beginning of each measurement. Since no charge control was applied, the charging time was made much longer than the ratio of battery capacity to the charging current. The resulting charging time was 10 h, with the charging current starting from 36 mA at the beginning of the process.

The transducer coil voltages measured in the first stage were processed in the following way.

**Figure 9:**
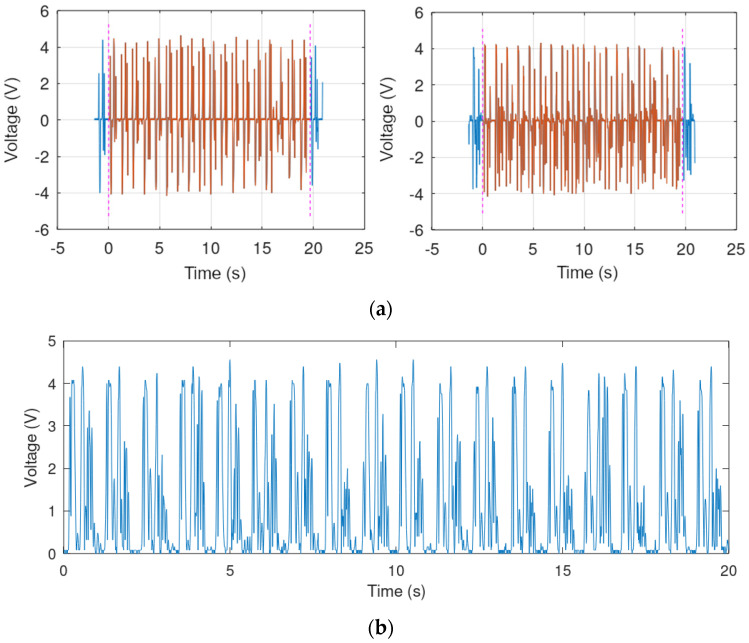
Coil voltage processing, Volunteer 4 walking with a speed of 3 km/h: (**a**) truncation of the waveform from either coil (blue waveform: signal recorded by the oscilloscope, red waveform: truncated signal), (**b**) combination of waveforms after their rectification.

(1) Outermost parts of the waveform were rejected and the waveform was truncated to an integer multiple of step length, as shown in Figure 9a.
(2) Waveforms from the two coils were combined in one waveform corresponding to the operation of the diode bridge present in the power processing circuit, with the use of the absolute value function, as shown in Figure 9b (function generator outputs are ground-referenced, which makes it impossible to generate two independent voltages corresponding to the two coils).

The processed waveforms were converted with the Tektronix ArbExpress software to a file format appropriate for the function generator. The function generator output was connected to the signal amplifier input. The output of the latter was connected to the power processing circuit via the connection board with the oscilloscope probe. The signal amplifier gain was adjusted by observing the waveform on the oscilloscope so as to obtain the same signal amplitude as recorded in reality. The waveform at the output of the function generator was repeated in a loop until the output converter voltage went off, testifying to the battery becoming discharged. The operation time was measured with a stopwatch from the moment of turning on the S1 switch in the power processing circuit till the “no power” detector sound signal was heard.

The following two cases of input voltage waveforms were selected to represent the best and the typical case scenarios.

**Figure 10:**
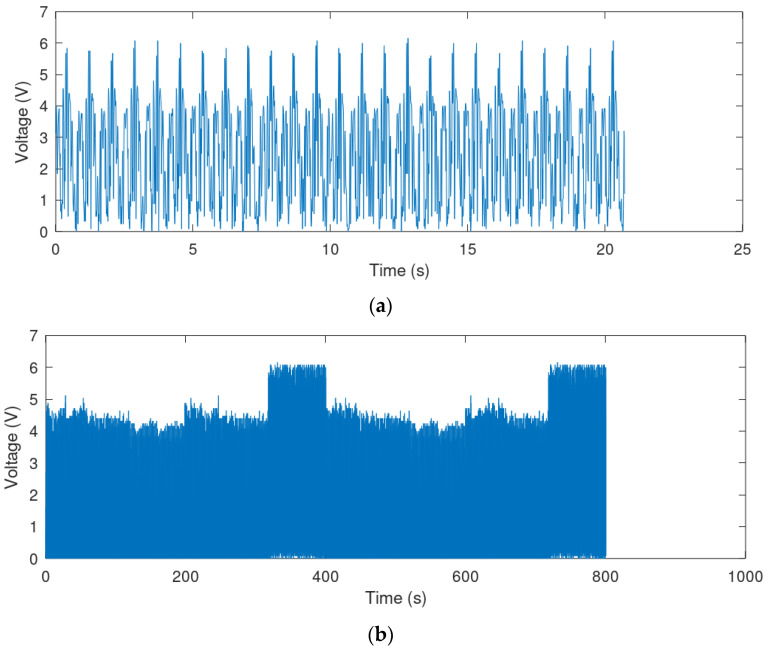
Voltage waveforms from the function generator: (**a**) the best case, (**b**) the typical case.

(1) The best case was chosen as the one providing the highest RMS value and amplitude of voltage; this was walking on flat with a speed of 8 km/h for Volunteer 4, trial 1. The waveform length for this case was 20.71 s (Figure 10a).
(2) The typical case was created by combining all the four studied cases in specific proportions (Table 1); the components are listed in Table 2, while their percentage shares are shown in Table 3. The total length of the combined waveform is 800.38 s (Figure 10b).

The reference case was one with the function generator disconnected, thus with only the battery supplying the load.

### 2.7. Ergonomics Assessment

In order to ensure a comprehensive evaluation of the developed solution, ergonomics assessment was also introduced to the methodology. The aim of this part of the tests was to verify whether the electromagnetic generator does not cause any burden to its potential user, i.e., mountain rescuer in this specific case. A new test methodology was designed which included the most common and characteristic activities performed by mountain rescuers during their rescue operations. The activities were selected on a basis of the consultation with mountain rescuers in order to ensure that simulated conditions will be close to real. The same procedure was applied in an earlier study to assess a system with photovoltaic generators [20]. Finally, five exercise stations were included in the experiment:

- uphill climbing simulation: an exercise on a climbing trainer at a speed of 40 steps/min, stride length of approx. 70 cm;
- simulation of providing first aid: an exercise in a squatting position;
- lifting simulation: an exercise with a 81 kg load on a lower lift;
- downhill walk simulation: walking down a treadmill at a speed of 3 km/h, with a 20% tilt;
- simulation of climbing a rope: an exercise with a 81 kg load on an upper lift.

**Figure 11:**
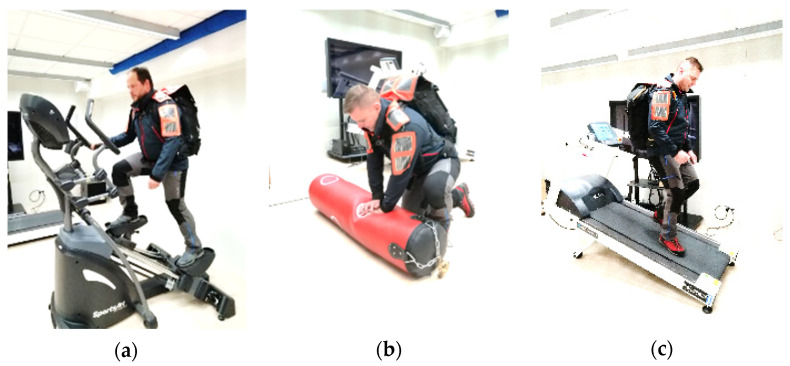
Mountain rescuer during ergonomic tests (**a**) climb walking, (**b**) squatting, (**c**) downhill walk [20].

Images of a volunteer undertaking the selected activities are presented in Figure 11. Two professional mountain rescuers were involved in the ergonomic tests. They both performed adopted activities lasting from 2 to 3 min at each station. At the end of the tests, the volunteers were asked to complete a survey questionnaire and additionally the interview was performed. In the questionnaire, mountain rescuers assessed the developed solution in terms of its ergonomics (e.g., ease of body movements, existence of harmful effects, compatibility with other equipment), as well as level of the technology acceptance (e.g., attitude to energy harvesters, usability of the developed solution). Each feature was evaluated with a 5-point scale, in which ‘1’ was the worst score and ‘5’ was the best one. In the interviews, additional comments from mountain rescuers were collected. A special attention in the discussion was paid on identification of eventual modifications to the developed solution that should be implemented in order to improve its usability.

### 2.8. Methodology Validation

The methodology was validated by first obtaining data from the transducer used by a volunteer in the field and second, processing them and performing measurements in laboratory using the electronic model, as described in Section 2.6. Due to the need of preserving consistent and invariant test conditions as well as to the long time necessary to discharge the battery by a realistic load, it was decided to limit the experiments to a walking activity in a flat terrain.

To enable the volunteer to keep a constant speed for an extended time without considerably decreasing the transducer power output, the velocity was reduced to 6 km/h; it was monitored using a GPS device. The activity was performed along two straight sections of a paved path with a total length of 1.3 km and an average slope of 0.69%. The distance was covered several times in both directions until the battery discharged. To reduce the duration of the activity, an Cellevia LP142817 Li-ion battery (Ropla Elektronik, Suchy Dwór, Poland) with a nominal capacity of 33 mAh was used, resulting in a discharge time of about 50 min with still the same load. In these conditions, system operating time was measured in the manner described in Section 2.5.

To obtain data necessary to run the electronic model, additional measurements were realized in the same environment and for the same activity type and velocity, but over short intervals so that transducer waveforms could be recorded using an oscilloscope with a sufficient time resolution. Five such recordings were made, processed and combined in accordance with Section 2.6.

**Figure 12:**
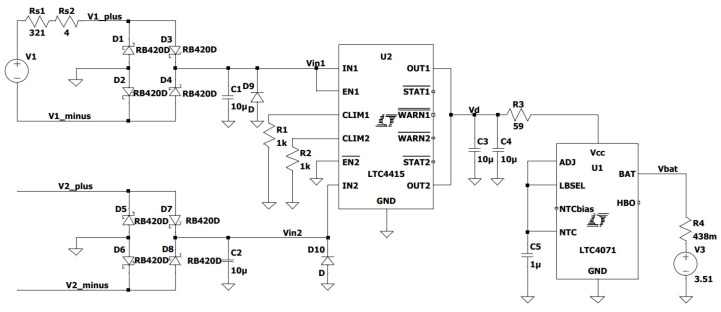
Simulation circuit for the methodology second stage validation.

In addition, a model for computer simulation was created, whose schematic is shown in Figure 12. The simulations were conducted in LTspice software (Analog Devices, Wilmington, MA, USA) using the models of LTC4415 and LTC4071 components available there. The battery was modelled with a voltage source (*V3* in Figure 12) with a series resistance set according to its datasheet. The voltage was adjusted according to the values measured for different battery states. All the other component parameters corresponded to manufacturer-provided data or to ones measured. The *Rs1* resistance of 321 Ω corresponds to a resistor located on the connection board and the *Rs2* resistance of 4 Ω is the output impedance of the signal amplifier, so that the total resistance equaled that of the Ampy-Move transducer coil (Section 2.2). The simulation circuit input voltage (the *V1* source in Figure 12) was generated from the exact file used by the function generator. The second input was left disconnected as in the real procedure.

## 3. Results

### 3.1. Generator Average Power in Real Conditions

**Figure 13:**
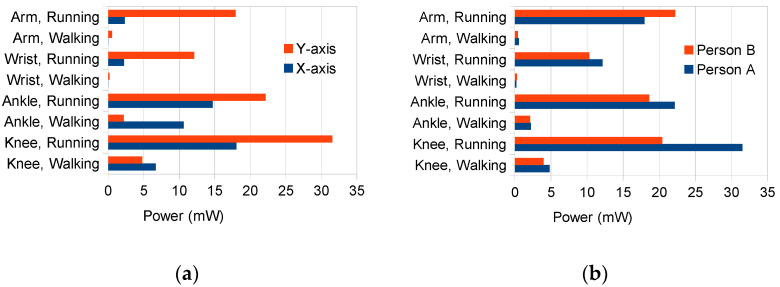
Measured average power levels for the Ampy-Move transducer loaded with the 330 Ω optimum load: (**a**) for two different axial orientations, (**b**) in the *Y* axis for two different persons.

Figure 13 presents measurement results of average power levels generated by the Ampy-Move transducer (per one coil) in real conditions when loaded with the 330 Ω resistive load. The *X* axis represents the horizontal direction of the generator, i.e., the one parallel to the motion direction, while the *Y* axis represents the vertical direction.

According to the results, the power level depends on the physical activity type (walking or running), the location of the generator and the generator’s axial orientation. For the dominant physical activity of a mountain rescuer, which is walking, it is the most beneficial to place the generator on the ankle in the *X* axis; then, the generated power can reach 10 mW per coil. However, this generator location may be inconvenient for the user. A power approximately two times lower is generated when placing the generator below the knee, in the Y direction, when the user walks (as it was done in the laboratory experiments). This location permits to avoid potential ankle injuries caused by loose generator fastening or a strain caused by its mass when walking for long distances with a high pace. It is worth mentioning that the location chosen is the most beneficial for a running activity.

### 3.2. Maximum Force as well as Step and Stride Lengths in Laboratory Conditions

**Figure 14:**
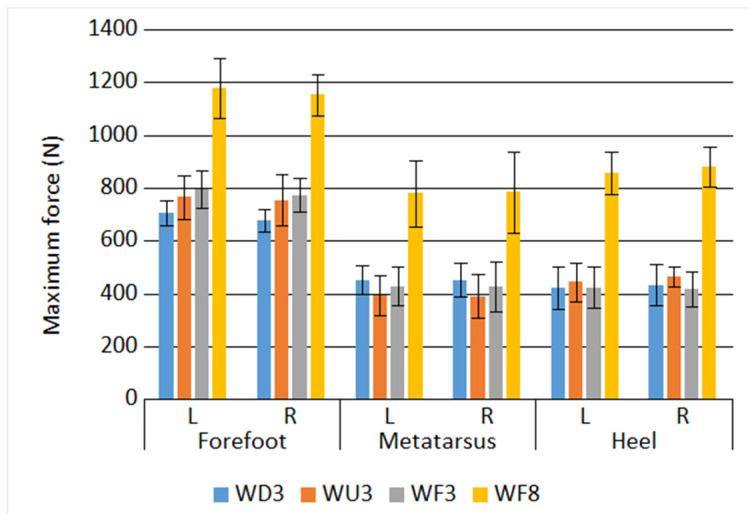
Maximum force generated during selected physical activities.

**Figure 15:**
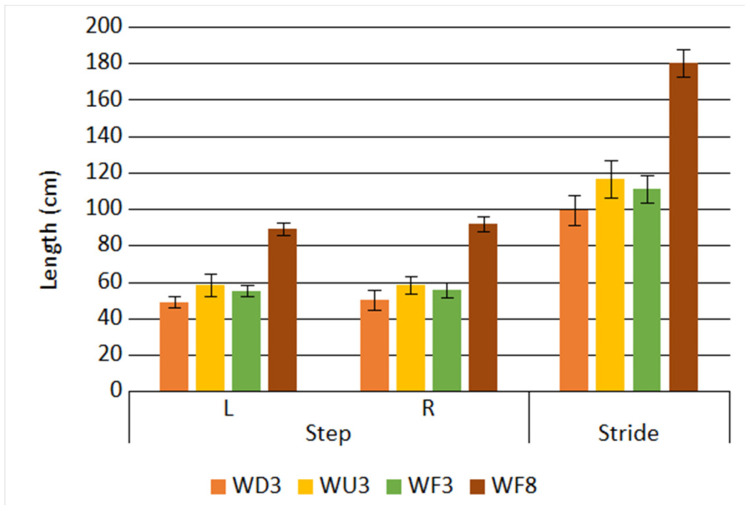
Step and stride during physical activities.

Measurement results of generated maximum forces and step and stride lengths are shown in Figure 14 and Figure 15.

According to these results, the maximum force is similar for both legs independent of the physical activity type. There are however substantial differences in maximum force values among the studied activity types themselves. The highest forces were generated for the MP8 activity, when the volunteer moved with the highest speed; the other activities (at the same speed of 3 km/h) did not show so high force variations. One can also observe that the highest force was generated on the forefoot in all activity types.

The longest steps and strides were observed for the WF8 activity and the shortest for the WD3 activity. For the same walking speed of 3 km/h, the stride length ranged from 100 cm to 120 cm, and it increased with the tilt angle.

### 3.3. Generator Characteristics in Laboratory Conditions

**Figure 16:**
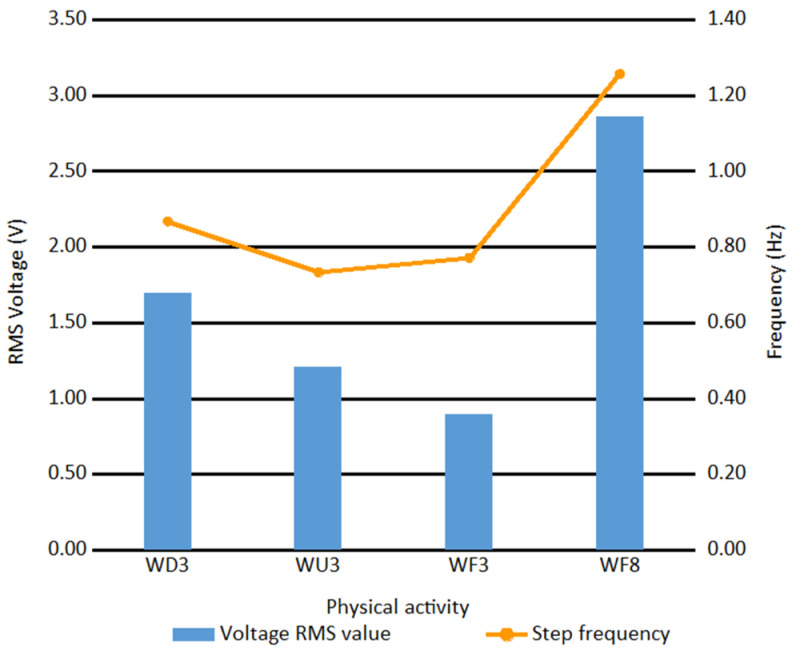
Generator output parameters for different physical activity types.

The RMS voltage values and frequencies of the fundamental components measured during the different activities are presented in Figure 16.

The obtained results show that the highest RMS voltage was achieved for the WF8 activity and the lowest for the WF3 activity. It means that motion speed is the main factor influencing the generated voltage RMS value. Moreover, downhill and uphill motions are beneficial as compared to the motion on flat: the RMS voltages were higher when a tilt was nonzero. One can also observe that for the WF3 activity, the frequency of steps was higher than for WU3, although the RMS voltage value was lower. It may mean that maximum force generated by a heel was important than the frequency of steps. The results show that both the physical activity type and the motion speed influence the efficiency of the EM generator used in the system. Basing on them, the WF8 activity was shown to be the most favorable from the point of view of energy.

### 3.4. Energy Harvesting System Efficiency

**Figure 17:**
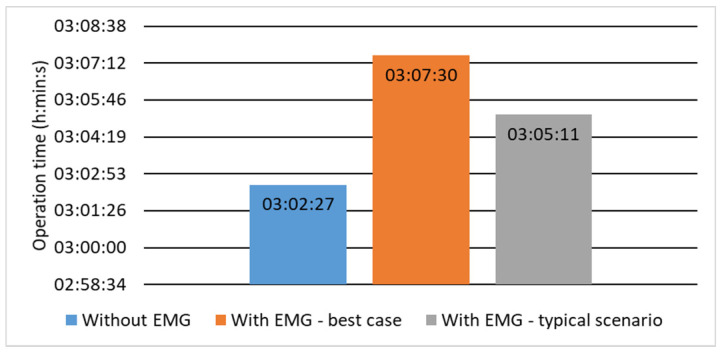
Active component operating time.

The results of the study on the energy harvesting system efficiency, expressed as the additional active component model operation time achieved by the application of the biomechanical energy transducer, are presented in Figure 17 for the two scenarios (the best and the typical one).

The obtained results show that the use of the electromagnetic transducer increased the operation time by 5 min and 3 s in the best case and by 2 min and 44 s in the typical case, which corresponds to 2.8% and 1.5%, respectively. As a result, the average charging current was 1 mA in the best case and 0.6 mA for the typical case. The additional charge was 1.5 mAh in the best case and 0.81 mAh in the typical case and additional energy was 7.5 mWh and 4.06 mWh, respectively.

### 3.5. Ergonomics Assessment

According to the mountain rescuers’ opinions, the energy harvesting system based on Ampy-Move installed on the leg, below the knee, did not hinder them during physical activities, did not impede their movements and did not cause excessive strain.

### 3.6. Methodology Validation Results

The measurement results obtained by performing physical activity in real terrain and by running the electronic model in the laboratory are compared in Table 4 for the five short-time attempts as described in Section 2.8. As the transducer’s output voltage is mainly dictated by the slowly varying battery voltage (with some influence of the input converter, see Figure 1), it is the average transducer current and the resulting total charge delivered that describe the power input. As the battery voltage has an effect on this charge, experiments were conducted after discharging the battery; the latter, however, became charged as a result of testing. For this reasons, model measurements were repeated after conducting their first round and letting the battery rest for ca. 1.5 h.

Although care was taken to keep an invariant speed and movement character, it proved difficult in the field, even over a short flat path in open terrain. This is revealed by the different average transducer currents in each attempt. On the other hand, a very good agreement of the order of 10% or higher was obtained between real terrain results and those yielded by the electronic model, irrespective of the state of the battery.

Table 4 also contains results obtained by computer simulation as described in Section 2.8 (for a rested battery, corresponding to a volage of 3.51 V). Although they correctly predict the transducer’s yield as to the order of magnitude, the discrepancies are clearly higher than for the electronic model. This follows from the implicit and non-linear character of the system’s characteristics discussed in Section 2.1. It should be noted that the waveform produced by the voltage source was exactly the same as the one delivered by the function generator, manufacturer-supplied device models were used for the integrated circuits and datasheet parameters were applied for the battery and the diodes. The discrepancy would become even higher if the output converter was added, for which a simulation model was unavailable. For these reasons, computer simulations were not used for long-duration tests.

An ultimate validation of the developed methodology should be based on the system operating time, which is also the basis for calculating the harvested energy as shown by Equation (6). These results are presented in Table 5. As explained in Section 2.8, in contrast to those in Table 4, they are based on different attempts: the real terrain result was obtained directly in a long-duration exercise, while the waveform reproduced by the electronic model was a concatenation of the waveforms yielded by the five short-duration exercises. It must therefore be expected for the discrepancy to increase. Nevertheless, the operating time provided by the electronic model is just by 13% longer than the real one. Both these results also suffer from errors arising from the difficulty in keeping a constant walking speed without the use of a running track.

## 4. Discussion

As demonstrated in Section 3.6, the devised testing methodology yields results consistent with those obtained in real terrain, both on the low and the high levels of abstraction (transducer current and system operating time, respectively). The accuracy of the order of 10% should be regarded as high considering that ensuring an identical movement velocity was not possible. Nevertheless, the high degree to which results at different attempts varied is an additional argument for standardized laboratory tests at Stage 1 of the methodology (as opposed to field test) to ensure identical conditions and result comparability, especially if different transducers are to be compared.

A time of 1.5 h was necessary to carry out a single physical activity for methodology validation as described in Section 2.8. On the other hand, just about 1 h was found sufficient to collect data for various activities and parameters per one volunteer according to the procedure presented in Section 2.5. These data can then reused for different system configurations (such as battery replacement or power processor redesign) or transducers, while preserving exactly the same conditions. With the standard approach based solely on in-field measurements, such changes would require the physical activity to be carried out again without any guarantee of identical test parameters.

Laboratory measurements can additionally provide valuable data on several parameters that are impossible or difficult to measure in the field, such as mechanical force or step length (Section 3.2).

On the other hand, based on results presented in Section 3.6, it must be concluded that computer simulations cannot provide as accurate estimates of the system performance as the devised two-stage procedure. This is because of the complex relationships existing between the different system parameters that additionally vary in time. In such a case, an electronic model connected to the system in place of the true transducer provided more accurate results while making it possible to limit the duration of physical activities carried out by human volunteers.

For what regards the particular energy harvesting system using the Ampy-Move transducer that served as the example to demonstrate the methodology, the energy yield was found to be lower than estimated in [21]. This is due to the unrealistic test conditions in [21]: motion type, frequency and amplitude. The Ampy-Move is therefore not suitable to supply the selected GPS receiver. However, it could still be useful for smart clothing active components with a lower power consumption, such as temperature or humidity sensors.

The results presented in Section 3.1 show that the type of the physical activity and the motion speed had an important influence on the RMS voltage generated by the Ampy-Move transducer. Moreover, the motion direction (tilt) for a constant motion speed significantly influenced the generated voltage. Hence, a proper composition of the typical motion scenario is crucial for a reliable estimation of a system’s efficiency in real conditions.

The proposed methodology provides more reliable evaluation results with less time load on volunteers than the techniques applied in other works. In [9,10,11,16,17,18,19,22], any physical activity would have to be repeated for a modified or different transducer, and converter efficiency is assumed to be constant. In [10], test duration was of just several seconds, making it impossible to include the influence of energy bank charging and discharging. The authors of [12,15,17,18,19] did not include any power converter in their analyses, while its efficiency considerably affects the energy ultimately delivered to the load. Moreover, no real physical activity was involved in [18].

## 5. Conclusions

In the article, an evaluation methodology of electromagnetic biomechanical ener-gy transducers for energy harvesting has been proposed, composed of two stages. The first one consists of standardized tests in a laboratory environment, but simulating several real physical activities for different parameters (such as motion speed). At the second stage, an electronic model built with an arbitrary function generator is used to simulate the operation of the energy transducer.

This approach has made it possible to perform repeated tests in the same condi-tions while imitating real human body movements. The required time load on volun-teers was limited to about one hour, which is considerably less than even a single effi-ciency measurement in the field. However, a basic measurements in real terrain were also conducted to prove the validity of the methodology.

Thanks to the possibility of repeating measurements over an arbitrary time period while preserving invariable test conditions, the proposed evaluation methodology can be applied to any other system employing an electromagnetic energy transducer as well as to compare different solutions with each other. It makes it possible to easily as-sess the ultimate performance indicators such as the system operating time and the total energy harvested, even over very long time intervals.

## Figures and Tables

**Table 1:** Selected physical activity variants.

Code	Speed (km/h)	Tilt (%)	Motion Type
WD3	3	15	Downhill
WU3	3	15	Uphill
WF3	3	0	On flat
WF8	8	0	On flat

**Table 2:** Typical case scenario components.

Motion Type	Speed (km/h)	Trial Number	Processed Waveform Duration (s)
Uphill	3	1	20.1
Uphill	3	2	19.4
Uphill	3	3	19.2
Downhill	3	1	19.7
Downhill	3	2	20.0
Downhill	3	3	20.2
On flat	3	1	19.4
On flat	3	2	21.0
On flat	3	3	20.2
On flat	3	4	18.9
Uphill	3	4	20.5
Uphill	3	1	20.1
Uphill	3	2	19.4
Downhill	3	1	19.7
Downhill	3	2	20.0
Downhill	3	3	20.2
On flat	8	1	20.7
On flat	8	2	20.7
On flat	8	3	21.6
On flat	8	4	19.2
Uphill	3	3	19.2
Uphill	3	4	20.5
Uphill	3	1	20.1
Downhill	3	1	19.7
Downhill	3	2	20.0
Downhill	3	3	20.2
On flat	3	1	19.4
On flat	3	2	21.0
On flat	3	3	20.2
On flat	3	4	18.9
Uphill	3	2	19.4
Uphill	3	3	19.2
Uphill	3	4	20.5
Downhill	3	1	19.7
Downhill	3	2	20.0
Downhill	3	3	20.2
On flat	8	1	20.7
On flat	8	2	20.7
On flat	8	3	21.6
On flat	8	4	19.2

**Table 3:** Shares of motion types in the typical case scenario.

Motion Type	Speed (km/h)	Overall Duration (s)	Share in the Scenario (%)
Uphill	3	237.48	29.7
Downhill	3	239.48	29.9
On flat	3	159.14	19.9
On flat	8	164.28	20.5

**Table 4:** Electric charge from the transducer as measured in real terrain and yielded by the electronic model.

Battery State	Attempt No.	Charge Delivered (mC)		Average Current (mA)			Error (%)	
		In-field	Electronic Model	In-field	Electronic Model	Computer Simulation	Electronic Model	Computer Simulation
Immediately after Discharge	1	11.56	12.34	0.547	0.584		6.7	
	2	9.72	10.35	0.473	0.504		6.6	
	3	12.43	13.26	0.612	0.652		6.7	
	4	11.55	12.89	0.602	0.672		11.6	
	5	9.72	10.13	0.474	0.494		4.3	
After First Test Round and Resting	1	11.56	10.14	0.547	0.480	0.391	−12.3	−28.3
	2	9.72	8.39	0.473	0.409	0.330	−13.7	−30.3
	3	12.43	11.01	0.612	0.541	0.448	−11.5	−26.7
	4	11.55	10.74	0.602	0.560	0.463	−7.0	−23.1
	5	9.72	8.55	0.474	0.417	0.333	−12.0	−29.6

**Table 5:** System operating time as measured in real terrain and yielded by the electronic model.

Test Environment	System Operating Time (mm:ss)
Real Terrain	56:19
Electronic Model	63:41

## Data Availability

The data presented in this study are available on request from the corresponding author.
